# Evaluation of relationships between onychomycosis and vascular diseases using sequential pattern mining

**DOI:** 10.1038/s41598-018-35909-z

**Published:** 2018-12-14

**Authors:** Chul Hwan Bang, Jae Woong Yoon, Hyun Ji Lee, Jun Young Lee, Young Min Park, Suk Jun Lee, Ji Hyun Lee

**Affiliations:** 1 Department of Dermatology, Seoul St. Mary’s Hospital, College of Medicine, The Catholic University of Korea, Seoul, Korea; 2 Department of Business Management, Kwangwoon University, Seoul, Korea

## Abstract

Onychomycosis (OM) is a common nail disease. Although controversial, vascular diseases are considered independent predictors of OM and vice versa. Sequential pattern mining (SPM) has not been previously used for statistical analysis in dermatology, but it is an efficient method for identifying frequent association rules in multiple sequential data sets. The aim of our study was to identify the relationship between OM and vascular diseases in the real world through a population-based study using SPM. We obtained population-based data recorded from 2002 to 2013 by the Health Insurance Research and Assessment Agency. Cases of vascular-related disease and OM were identified using the diagnostic codes of the International Classification of Diseases 10th Revision, version 2010. SPM measures were based on comorbidity and duration values. We estimated 3-year risk for progression from OM to vascular disease and vice versa using logistic regression. Patients with varicose veins and peripheral vascular disease had higher OM comorbidity (comorbidity: 1.26% and 0.69%, respectively) than did those with other vascular diseases. Patients diagnosed with varicose veins and peripheral vascular disease were diagnosed with OM after 25.50 and 55.10 days, respectively, which was a shorter duration than that observed for other diseases. Patients with OM were at higher risk for peripheral vascular disease (adjusted odds ratio (aOR) 1.199 [95% confidence interval (CI) 1.151–1.249]) and varicose veins (aOR 1.150 [95% CI 1.063–1.245]). Patients with peripheral vascular disease (aOR 1.128 [95% CI 1.081–1.177]) were at higher risk for OM, while patients with varicose veins had no significant risk for OM. Careful consideration of varicose veins or peripheral vascular disease is required for proper management of comorbidities in patients with OM.

## Introduction

Onychomycosis (OM) is a common condition, accounting for up to half of all reported nail diseases^1,2^. The estimated prevalence of OM in the general population ranges from 2 to 11%^3^. OM is associated with increasing age, gender, chronic diseases, smoking, immune dysfunction, and vascular diseases^3–5^. Among these risk factors, vascular diseases such as chronic vascular insufficiency and peripheral arterial disease are considered independent predictors of OM^4–6^. Conversely, OM could be considered a warning sign of cardiovascular disease^1^. However, according to one study, the prevalence of venous insufficiency was increased in patients with OM, but the prevalence of peripheral arterial disease was not increased^7^. The association of vascular disease with OM remains unclear; thus, large-scale studies of their relationship are needed.

Sequential pattern mining (SPM), among other association rule mining methods, is an efficient method for finding frequent association rules in multiple sequential data sets^8,9^. SPM is based on analyzing transaction histories at the point of sale in the retail industry, but it is also useful for identifying signs of complications when multiple diseases are present in a patient’s medical records.

The aim of our study was to identify the relationship between OM and vascular disease in the real world through a population-based study using SPM.

## Methods

### Data source

We obtained population-based data recorded from January 1, 2002 to December 31, 2013 by the Health Insurance Research and Assessment Agency (HIRA), which is affiliated with the National Health Insurance Corporation (NHIC) of Korea. The HIRA database contains comprehensive health-related information about the population of Korea and comprises an eligibility database, health examination database, medical care institution database, and medical treatment database. The entire Korean population is covered by the NHIC. In 2013, 97.2% (n = 49,989,620) of the population was covered by the NHIC database, and the remaining 2.8% (n = 1,458,871) of the population was covered by the medical aid system^10^. In addition, the HIRA provides a standardized, stratified one million-person sample population (n = 1,116,789) from the NHIC database.

Our study data were extracted from the National Health Insurance Corporation (NHIC) claim database. All data were anonymized and provided to the researcher except for the patient’s personal information. This study was approved by the Institutional Review Board of the Korean NHIC (no. NHIS-2017-1-002). The study design was approved by the Ethics Committee of Seoul St. Mary’s Hospital, the Catholic University of Korea (approval no. KC16EISE0923) and followed all relevant principles of the Declaration of Helsinki. Informed consent was not necessary due to the nature of our study.

### Identification of vascular disease and OM

We used the diagnostic codes of the International Classification of Diseases 10th Revision (ICD-10), version 2010. Patients with OM were defined as those who were diagnosed with B35.1 ICD-10 code. Patients with vascular-related disease were identified by vascular disease-related ICD-10 codes (primary hypertension [I10], angina pectoris [I20], cerebral infarction [I63], other peripheral vascular diseases [I73], varicose veins [I83], and hemorrhoids [I84]). To investigate the incidence of vascular diseases newly occurring in OM and vice versa, we excluded patients who had been diagnosed with vascular disease codes or OM at least once in 2002 to 2003.

### SPM

We used SPM to examine relationships between diseases caused by time differences, and SPM measures were based on confidence and duration values. Confidence was defined as the conditional probability of a sequential pattern^9^, and duration was the average occurrence time of the sequential pattern. In this analysis, gender, ICD-10, age, and date were used as variables to identify the sequential pattern of the disease using statistical software SAS Enterprise Miner version 13.2 (SAS Institute, Cary, NC, USA).

### Statistical analysis

To determine whether OM occurred more frequently in patients with each vascular disease and whether each vascular disease occurred in patients with OM, we established a cohort of patients diagnosed from 2010 to 2013. We excluded patients who were diagnosed with OM or each vascular disease before 2010. We compared the logistic regression results with the occurrence of each vascular disease in patients with new OM and the occurrence of each vascular disease in the control group. Gender and age adjusted odds ratios (aORs) and 95% confidence intervals (CIs) were calculated using multivariable logistic regression modeling. All data were analyzed using SAS Enterprise Miner version 13.2 (SAS Institute, Cary, NC, USA). A value of P < 0.05 was considered statistically significant.

## Results

Table 1 shows the frequencies of patients with OM, hypertension, angina pectoris, cerebral infarction, peripheral vascular disease, varicose veins, and hemorrhoids by gender and age. Based on the results for each code, the total number of male patients was 88,818, and the total number of female patients was 77,548. The number of patients aged 20 to 64 years was 117,781 (70.80%), the number of patients older than 65 years was 46,094 (27.71%), and the number of patients aged 0 to 19 years was 2,491 (1.50%). Hypertension showed the highest frequency (55,514), followed by OM (47,275) and hemorrhoids (22,672).

**Table 1:** Frequencies of patients with diagnostic codes for onychomycosis, primary hypertension, angina pectoris, cerebral infarction, other peripheral vascular diseases, varicose vein and hemorrhoids by gender and age.

Disease	Gender	Frequency	Age	Frequency	Total
Onychomycosis	Male	25,423	0~19	464	47,275
			20~64	20,396	
			Over 65	4,563	
	Female	21,852	0~19	791	
			20~64	17,901	
			Over 65	3,160	
Primary Hypertension	Male	29,177	0~19	43	55,514
			20~64	18,014	
			Over 65	11,120	
	Female	26,337	0~19	156	
			20~64	19,879	
			Over 65	6,302	
Angina pectoris	Male	8,046	0~19	44	16,222
			20~64	4,745	
			Over 65	3,257	
	Female	8,176	0~19	69	
			20~64	5,666	
			Over 65	2,441	
Cerebral infarction	Male	8,047	0~19	14	15,544
			20~64	2,736	
			Over 65	5,297	
	Female	7,497	0~19	15	
			20~64	3,505	
			Over 65	3,977	
Other peripheral vascular diseases	Male	4,942	0~19	47	7,713
			20~64	3,055	
			Over 65	1,840	
	Female	2,771	0~19	44	
			20~64	1552	
			Over 65	1,175	
Varicose veins	Male	915	0~19	6	1,426
			20~64	785	
			Over 65	124	
	Female	511	0~19	6	
			20~64	377	
			Over 65	128	
Hemorrhoids	Male	12,268	0~19	379	22,672
			20~64	10,557	
			Over 65	1,332	
	Female	10,404	0~19	413	
			20~64	8,613	
			Over 65	1,378	

**Figure 1:**
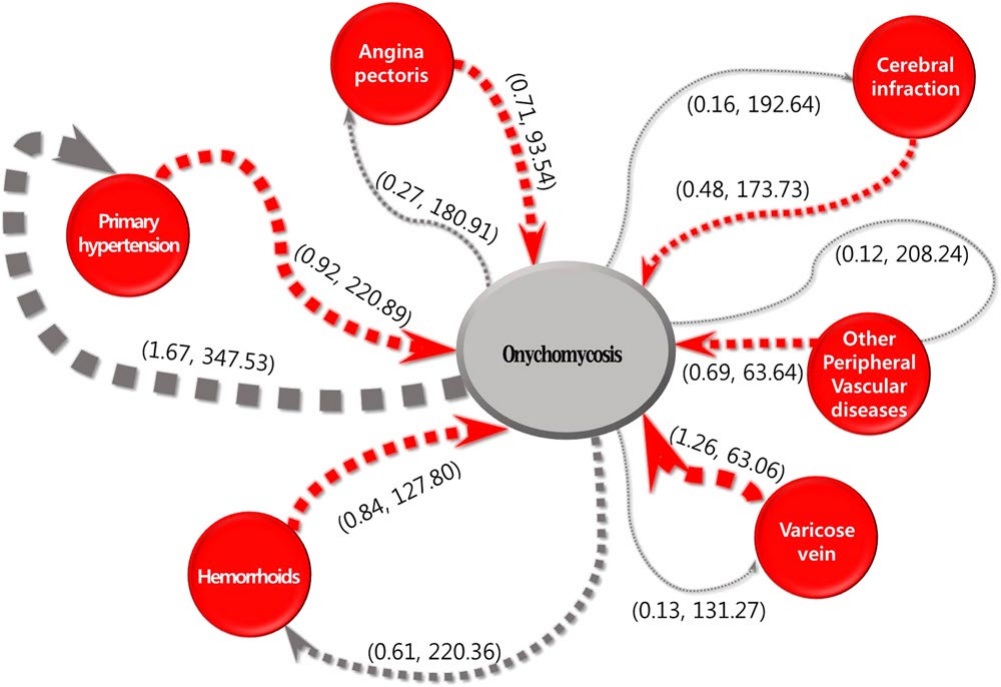
Confidences and onset durations for sequence patterns between onychomycosis and primary hypertension, angina pectoris, cerebral infarction, other peripheral vascular diseases, varicose veins and hemorrhoids. The first number in parentheses indicates the confidence (%), and the second number indicates onset duration (days).

Figure 1 shows sequence patterns between diseases and comorbidities, as well as the durations extracted through SPM. Parentheses indicate the data for each disease. The comorbidity association rule hypertension ⇒ OM indicates the percentage of patients with OM among patients with hypertension^11^. The duration refers to the sequence (average number of days) between diseases. A comorbidity of 1.67% for the association of OM ⇒ hypertension was the highest value of all association rules, followed by values of 0.61%, 0.27%, 0.16%, 0.13% and 0.12% for the association rules of hemorrhoids, angina, cerebral infarction, varicose veins, and peripheral vascular disease, respectively. The duration 131.27 days for the association rule of OM ⇒ varicose veins was the shortest duration, followed by 180.91, 192.64, 208.24, 220.36, and 347.53 days for the association rules of angina, cerebral infarction, peripheral vascular disease, hemorrhoids, and hypertension, respectively. A comorbidity of 1.26% for the association rule of varicose veins ⇒ OM was the highest value for all association rules, followed by 0.92%, 0.84%, 0.71%, 0.69% and 0.48% for the associations of hypertension, hemorrhoids, angina, peripheral vascular disease and cerebral infarction, respectively. The duration of 25.50 days for the association rule of varicose veins ⇒ OM was the shortest duration, followed by 55.10, 108.80, 133.66, 169.53, and 173.31 days for the association rules of peripheral vascular disease, angina, hemorrhoids, cerebral infarction, and hypertension, respectively.

**Figure 2:**
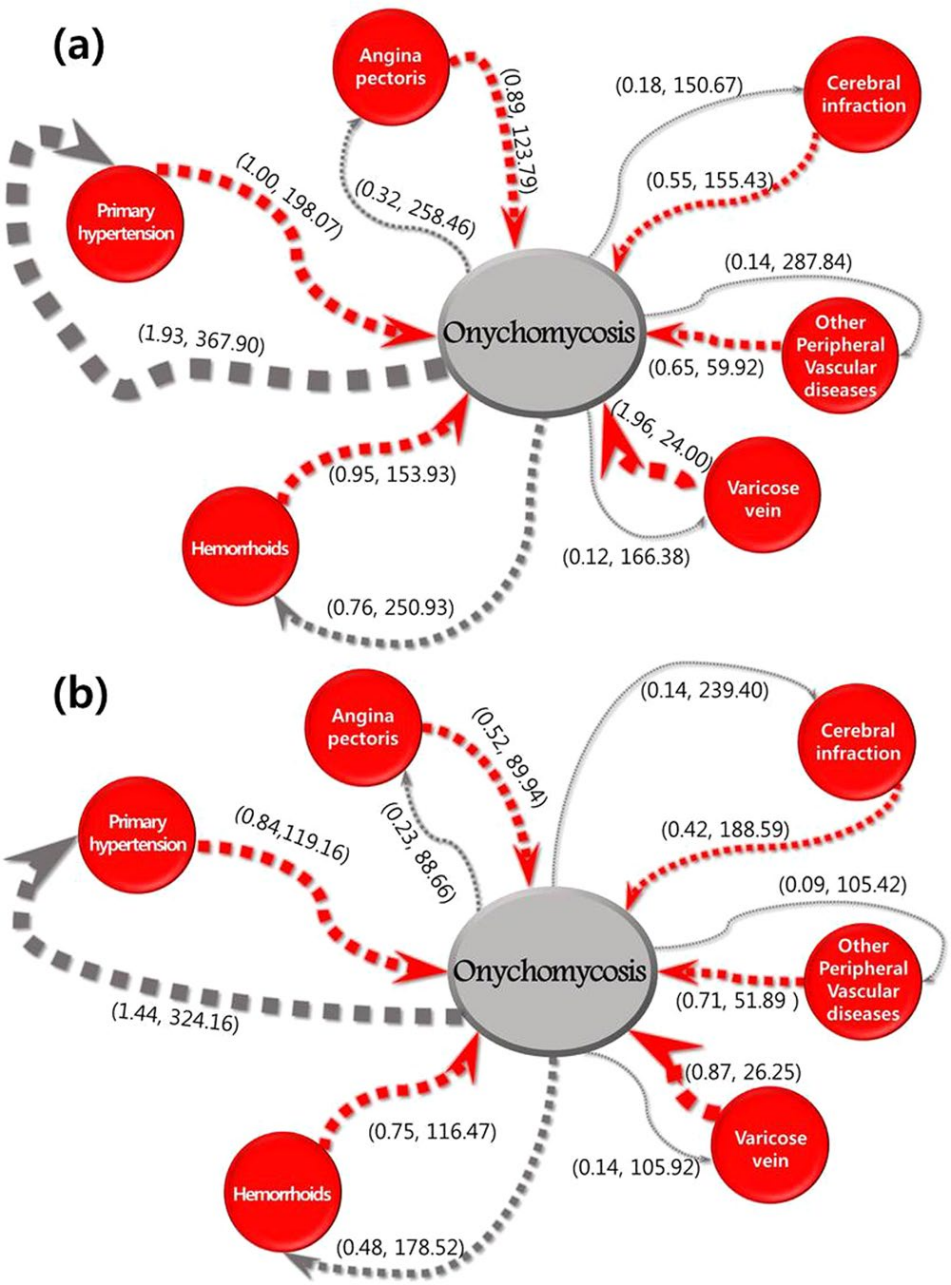
Confidences and onset durations for sequence patterns between onychomycosis and each vascular disease in (**a**) males and (**b**) females. The first number in parentheses indicates the confidence (%), and the second number indicates onset duration (days).

Figure 2 illustrates rules and their confidences and durations based on gender. For males (Fig. 2a), a comorbidity of 1.93% for the association rule of OM ⇒ hypertension was the highest value. The duration of 150.67 days for the association rule of OM ⇒ cerebral infarction was the shortest duration, followed by 166.38, 250.93, 258.46, 287.84, and 367.90 days for the association rules of varicose veins, hemorrhoids, angina, peripheral vascular disease, and hypertension, respectively. The comorbidity of 1.96% for the association rule of varicose veins ⇒ OM was the greatest value. The duration of 24.00 days for the association rule of varicose veins ⇒ OM was shortest duration, followed by 59.52, 123.79, 153.93, 155.43, and 198.07 days for the association rules of peripheral vascular disease, angina, hemorrhoids, cerebral infarction, and hypertension, respectively.

Among females (Fig. 2b), the comorbidity of 1.44% for the association rule of OM ⇒ hypertension was the highest value. The duration of 88.66 days for the association rule of OM ⇒ angina was the shortest duration. The comorbidity of 0.87% for the association rule of varicose veins ⇒ OM was the highest value. The duration of 26.25 days for the association rule of varicose veins ⇒ OM was shortest duration, followed by 51.89, 89.94, 116.47, 119.16 and 188.59 days for the association rules of peripheral vascular disease, angina, hemorrhoids, hypertension and cerebral infarction, respectively.

Table 2 shows the aORs and CI for comorbidities. Patients with OM were at higher risk for peripheral vascular disease (aOR 1.199 [95% CI 1.151–1.249]) and varicose veins (OR 1.150 [95% CI 1.063–1.245]). The risks of hypertension and hemorrhoids decreased by 7.4% (95% CI 0.865–0.959) and 8.4% (95% CI 0.882–0.852), respectively. No significant differences were observed in the risk of angina and cerebral infarction in patients with OM.

**Table 2:** Risk of onychomycosis for each vascular disease and vice versa.

	3-year risk of each vascular disease in patients with OM	OR (95% CI)	P		3-year risk of OM in patients with vascular disease	OR (95% CI)	P
**Hypertension**							
Controls	0.076% (62,276/818,743)	Reference		Controls	0.061% (49,120/802,664)	Reference	
OM	0.071% (3,694/52,137)	0.926 (0.895–0.959)	<0.0001	Hypertension	0.069% (4,481/65 405)	1.128 (1.093–1.165)	<0.0001
**Angina pectoris**							
Controls	0.024% (21,793/909,004)	Reference		Controls	0.07% (66,039/944,945)	Reference	
OM	0.025% (1,643/67,124)	1.021 (0.971–1.075)	0.412	Angina pectoris	0.072% (1,642/22,834)	1.031 (0.980–1.085)	0.236
**Cerebral infarction**							
Controls	0.015% (13,710/912,852)	Reference		Controls	0.071% (68,031/956,751)	Reference	
OM	0.014% (958/68,709)	0.927 (0.868–0.991)	0.25	Cerebral infarction	0.063% (890/14,204)	0.873 (0.816–0.935)	<0.0001
**Other peripheral vascular disease**							
Controls	0.033% (29,871/907,277)	Reference		Controls	0.069% (64,451/936,629)	Reference	
OM	0.039% (2,587/65,978)	1.199 (1.151–1.249)	<0.0001	Peripheral vascular disease	0.077% (2,417/31,417)	1.128 (1.081–1.177)	<0.0001
**Varicose veins**							
Controls	0.008% (7,747/919,540)	Reference		Controls	0.072% (69,313/969,680)	Reference	
OM	0.01% (675/69,750)	1.150 (1.063–1.245)	0.001	Varicose veins	0.068% (552/8,079)	0.953 (0.873–1.039)	0.273
**Hemorrhoids**							
Controls	0.048% (43,964/909,631)	Reference		Controls	0.07% (64,529/926,999)	Reference	
OM	0.045% (2,950/66,335)	0.916 (0.882–0.952)	<0.0001	Hemorrhoids	0.059% (2,672/45,601)	0.832 (0.799–0.866)	<0.0001

Patients with hypertension (OR 1.128 [95% CI 1.093–1.165]) and peripheral vascular disease (OR 1.128 [95% CI 1.081–1.177]) were at higher risk for OM. The risk of OM was decreased by 12.7% (95% CI 0.816–0.935) in patients with cerebral infarction and 16.8% (95% CI 0.799–0.866) in patients with hemorrhoids. No significant differences were observed in the risk of OM in patients with angina and those with varicose veins.

## Discussion

We investigated the comorbidity between OM and vascular diseases using SPM. We also investigated the onset duration from OM to each vascular disease and vice versa. In our study, patients with OM were at significantly increased risk of varicose veins and peripheral vascular disease. Furthermore, the onset duration of OM in patients with varicose veins or peripheral vascular disease was relatively short compared to that of other vascular diseases.

The comorbidity of OM was highest (1.26%) in patients with varicose veins. The percentage of new onset OM in patients with vascular disease and vice versa was defined as confidence. Since the value of the “confidence” parameter is synonymous with “comorbidity” in epidemiology, we also regarded confidence as comorbidity^9^. Because chronic venous insufficiency including varicose veins can lead to toenail OM, our results are consistent with those of previous studies^6,7,12^. In previous studies, 36.1% and 35.7% of chronic venous insufficiency patients had OM^5,6^. However, these previous studies were cross-sectional and relatively small (36 and 42 patients). Since our study used population-based standard samples (n = 1,116,789) and individuals with previously diagnosed vascular disease or OM were excluded, the comorbidity of OM was different from that in previous studies. Our results can be interpreted as the probability of each vascular disease occurring in patients with OM.

Because SPM was performed to estimate the disease probability, logistic regression was used to investigate the 3-year risk of OM for each vascular disease and vice versa. Among patients with OM, the risk of varicose veins was increased by 15% compared with that among controls. However, the OM risk among patients with varicose veins was not significantly different from that among controls. These results may be attributed to the diagnosis of OM before the diagnosis of varicose veins. Kulac, *et al*.^6^. suggested that OM could be an early finding in some patients with chronic venous insufficiency, consistent with our results.

A duration of 25.5 days was required for patients with varicose veins to develop OM. Progressive microangiopathic changes in nail capillaries are thought to contribute to the occurrence of OM^6^. According to Mosmiller, *et al*.^13^, lymphocyte counts were significantly lower in patients with severe chronic venous insufficiency than in those with mild insufficiency; moreover, the total WBC count was not significantly different between the two groups. Karahan *et al*. also reported similar data^14^. Lower lymphocyte counts can decrease cellular immunity, and immunosuppression and decreased cellular immunity are well-known predisposing factors for OM^15^. Therefore, OM occurs more rapidly in chronic venous sufficiency than in other vascular diseases.

Peripheral vascular disease (ICD-10 code I73) includes artery spasms, intermittent claudication and Raynaud syndrome and is synonymous with peripheral artery disease^16^. In our study, peripheral vascular disease exhibited 0.69% comorbidity with OM, which was lower than that of varicose veins. This difference in comorbidity with varicose veins may be due to the intensive, intermittent insufficiency of peripheral vascular disease. By contrast, varicose vein or chronic venous insufficiency is sustained. Peripheral vascular diseases, except for asymptomatic peripheral vascular disease, are more likely to be treated before microangiopathic changes in nail capillaries induce OM. The time to onset of OM was 55.1 days in patients with peripheral vascular disease, and more time was required for OM than for varicose veins to develop. Once microangiopathic changes have occurred in nail capillaries, OM manifests in patients with peripheral vascular disease as varicose veins. The onset duration of OM in patients with peripheral vascular disease is longer than that in patients with varicose veins because microangiopathic changes in peripheral vascular disease occur more intermittently than those in varicose veins.

The correlation between OM and peripheral vascular disease is controversial^1,7^. In our study, compared with controls, patients with OM showed a 19.9% increased risk of peripheral vascular disease. Patients with peripheral vascular disease also exhibited a 12.8% greater risk of OM than did controls. However, in patients with OM, the probability of varicose veins was 0.13%, and the probability of peripheral vascular disease was only 0.12%. In contrast to Fukunaga *et al*.^1^, concluding that OM is a warning sign for peripheral vascular disease or varicose veins is difficult.

For hypertension or cerebral infarction, the significant changes in OM risk appear to be related to the frequency of hospital visits. In our study, the risk of OM was increased by 12.8% in patients with hypertension and decreased by 12.7% in patients with cerebral infarction. We could not find a report showing that OM was associated with hypertension or cerebral infarction^3,4,15^. Patients with hypertension visit the clinic regularly and have many opportunities to consult with their physician about their condition. Therefore, the diagnosis rate of OM among patients with hypertension is increased. However, the rate of OM in patients with cerebral infarction is decreased because they have more difficultly visiting their physician than do patients with other diseases. Further research on the risk of OM in patients with hypertension or cerebral infarction is needed.

The risk of hemorrhoids in patients with OM and vice versa was significantly decreased in our study. These results may have been observed because the epidemiologic spectra of the two diseases are different. Hemorrhoids are more frequent in higher socioeconomic status groups, whereas OM is more frequent in lower socioeconomic status groups^17,18^. Diabetes mellitus is a well-known risk factor for OM, but it does not increase the risk of hemorrhoids^17,19^. We suggest that the risk of comorbidity of these two diseases is decreased because the patient spectrum is different.

In our study, men took more time to visit the hospital due to OM than did women. Although OM is more prevalent in men than in women, men visit the hospital later than do women. The difference between the incidence of OM in men and women might be a reflection of the degree to which individuals are concerned about the appearance of their nails^20^. Because complications can occur when OM is left untreated in patients with diabetes or chronic diseases, physicians should be more concerned about the presence of OM in male patients.

This study has several limitations. First, because data were collected from the NHIC claims database, other potentially relevant data, including smoking history, family history and exercise history, were unavailable. Therefore, we focused on varicose veins and peripheral vascular disease, which were considered independent predictors of OM. Second, this study was a retrospective cohort study. Another limitation was the possibility of underestimation of diagnoses. Underestimation could occur when the symptoms of each disease were mild and because patients might not visit their physicians. Nevertheless, the strength of this study is the inclusion of data with standardized, stratified information from a one million-person sample population.

In conclusion, patients with OM patients were at increased risk for peripheral vascular disease and varicose veins; additionally, patients with peripheral vascular disease were at increased risk for OM. Careful consideration of varicose veins or peripheral vascular disease is required for the proper management of comorbidities in patients with OM.
